# Ileocecal and Small Bowel Involvement Are Independently Associated with Inferior Survival Despite Complete Cytoreduction in FIGO IIIC–IV Tubo-Ovarian and Primary Peritoneal Carcinoma

**DOI:** 10.1245/s10434-026-19485-5

**Published:** 2026-04-12

**Authors:** Ingo B. Runnebaum, Angela Kather, Clara Evangelia Goerdt, Huyen Thi Thanh Nguyen, Davit Bokhua

**Affiliations:** 1 Department of Gynecology and Reproductive Medicine, Jena University Hospital, Friedrich- Schiller-University Jena, Jena, Germany; 2 RU21 GmbH, Jena, Germany; 3 Zentrum für Alternsforschung Jena - Aging Research Center Jena, Jena, Germany

**Keywords:** Ovarian neoplasms, Peritoneal neoplasms, Debulking, Surgical, Small intestine/surgery, Ileocecal valve/surgery, Postoperative complications, Anastomotic leak, Precision oncology, Neoadjuvant therapy, Patient care team

## Abstract

**Background:**

Complete macroscopic tumor resection is the strongest prognostic factor in advanced epithelial tubo-ovarian and primary peritoneal cancer, yet benefit from maximum-effort cytoreductive surgery may vary with tumor distribution and site-specific resections. We aimed to identify predictors of long-term survival, considering postoperative morbidity, to inform preoperative stratification.

**Patients and Methods:**

This study had a retrospective single-center cohort of 302 all-comers patients with International Federation of Gynecology and Obstetrics/American Joint Committee on Cancer (FIGO/AJCC) IIIC–IV epithelial tubo-ovarian or primary peritoneal cancer undergoing maximum-effort cytoreductive surgery in a European Society of Gynecological Oncology (ESGO)-certified high-volume tertiary referral center (2006–2021). Major complications (Clavien–Dindo ≥ IIIb) were analyzed using multivariable logistic regression; progression-free and overall survival (PFS/OS) using Cox regression. Subgroup analyses explored site-specific bowel resections.

**Results:**

Complete resection was achieved in 259 (85.8%) patients, requiring high surgical complexity in 221 (73.2%, Surgical Complexity Score ≥ 8). Intestinal segment resections were performed in 71.5% of patients, including ileocecal resection in 24.5%. Large bowel resection (OR 2.708, *p* = 0.002) significantly increased major postoperative morbidity. Anastomotic leakage occurred in 6.0%, independent of transitory stoma formation (*p* = 0.759). Small bowel resection independently predicted impaired long-term survival (3-year OS 31.8% versus 57.0%, *p* < 0.001). Ileocecal resections were associated with poorest outcome (3-year OS 24.2%, *p* < 0.001). Complete macroscopic resection remained prognostically beneficial. Neoadjuvant chemotherapy (13.9%) and high surgical complexity did not negatively affect long-term survival.

**Conclusions:**

Ileocecal and small bowel involvement are independently associated with inferior survival despite complete cytoreduction in FIGO IIIC–IV disease. These findings support preoperative triage and counseling by the multidisciplinary tumor board, with selective consideration of primary systemic therapy with planned interval cytoreduction for extensive small bowel/ileocecal disease, particularly in frail or complex patients.

**Supplementary Information:**

The online version contains supplementary material available at 10.1245/s10434-026-19485-5.

Epithelial tubo-ovarian and primary peritoneal cancer remain the most lethal gynecologic malignancies, with two-thirds diagnosed at International Federation of Gynecology and Obstetrics (FIGO) stage IIIC/IV, and limited preventive and screening options.^1–5^ While primary cytoreductive surgery followed by platinum-based chemotherapy is the standard of care, survival is highly dependent on achieving complete macroscopic tumor resection.^6–9^ Accordingly, national and international guidelines have endorsed primary maximum-effort debulking surgery (PDS) for advanced disease, including multivisceral resections where necessary.^10^ Complexity of the applied procedures can be assessed using the validated Surgical Complexity Score (SCS) ^11^ to enable association with postoperative morbidity and prognosis.

However, this aggressive surgical approach is associated with substantial perioperative morbidity and requires coordinated multidisciplinary management. Not all patients equally benefit from maximal surgical effort despite complete tumor removal, and current stratification tools are limited.^12,13^ There is increasing evidence that surgical complexity itself can impact survival,^14,15^ likely due to delayed chemotherapy or persistent postoperative sequelae. Thus, precise patient selection and individualized preoperative risk assessment are crucial for optimal outcomes.^16^

In selected cases where a complete resection seems unlikely or the surgical risks are prohibitive, neoadjuvant chemotherapy (NACT) followed by interval debulking surgery (IDS) has been validated as noninferior approach in terms of overall survival.^12–15^

To address this challenge, we conducted a comprehensive analysis in a real-world all-comers cohort of patients with FIGO/ American Joint Committee on Cancer (AJCC) stage IIIC/IV epithelial ovarian, fallopian tube, or primary peritoneal cancer, uniformly treated by primary maximum effort cytoreductive surgery with the goal of complete macroscopic resection. These stages represent the most common and prognostically unfavorable subset of this type of cancer and often require technically demanding multivisceral resections.^16,17^

We hypothesized that specific tumor spread patterns—such as small bowel or ileocecal involvement and high surgical complexity—impact on perioperative risk and long-term survival. We sought to contribute information for individualized treatment decisions by identifying patients who may benefit most from extensive surgery and those who may require adapted strategies, such as NACT-IDS.

## Patients and Methods

### Study Setting

This retrospective single-center cohort study was conducted at the Department of Gynecology and Reproductive Medicine, Jena University Hospital, Germany, and included patients treated for advanced epithelial ovarian, fallopian tube, or primary peritoneal cancer with FIGO/AJCC stage IIIC–IV (i.e., pT3c N0/N1 M0 to any T any N M1 a/b) with the concept of maximum effort cytoreductive surgery to reach macroscopically complete tumor resection. The center was accredited by the German Cancer Society (DKG) as a Gynecological Cancer Center and by the European Society of Gynecological Oncology (ESGO) as a certified Center for Advanced Ovarian Cancer Surgery, fully adhering to all ESGO Quality Indicators,^16,17^ the German S3 Guidelines, and the National Comprehensive Cancer Network (NCCN) Clinical Practice Guidelines in Oncology. During the study period, it served as a tertiary referral center for Thuringia (population of approximately 2 million) with surgical leadership provided by IBR. The primary aim was to evaluate the impact of clinical and surgical characteristics, including age, tumor spread, and surgical complexity on postoperative morbidity and long-term oncologic outcomes.

This study was performed in line with the Strengthening The Report Of Cohort Studies in Surgery (STROCSS) 2024 guidelines and the REporting of studies Conducted using Observational Routinely collected health Data (RECORD) statement.^18,19^

### Patient Population

Consecutive patients treated for suspected epithelial ovarian, tubal, or peritoneal cancer between January 2006 and March 2021 were screened for inclusion on the basis of the following criteria: FIGO 2014/AJCC 2018 stage ≥ IIIC, histologically confirmed diagnosis, primary cytoreductive surgery aiming for complete resection, receipt of standard systemic therapy, and availability of complete clinical and follow-up data. Staging was retrospectively updated according to FIGO 2014, harmonized with AJCC (8th edition, 2018) and UICC (8th edition, 2017/2018) tumor nodes metastasis (TNM) systems. All cases underwent preoperative multidisciplinary team (MDT) review. Triage to PDS versus NACT–IDS considered disease distribution with specific attention to small bowel and ileocecal involvement, patient fitness, and the likelihood of complete macroscopic cytoreduction based on contrast-enhanced computed tomography (CT). Diagnostic laparoscopy was selectively performed when resectability remained uncertain, and a more systematic laparoscopy-based approach was adopted after the study period. Patients who received 3–6 cycles of NACT externally and were referred for surgery within 6 months of diagnosis were included. Exclusion criteria were stages < IIIC, missing data, non-epithelial malignancy, previous or concomitant abdominal cancer, or concomitant severe comorbidities that contraindicate cytoreductive surgery or chemotherapy or any deviation from standard primary treatment protocols.

### Surgical Procedures and Perioperative Management

All operations were performed by a dedicated gynecological oncology team (at least one board-certified consultant scrubbed in in every case, with IBR serving as primary surgeon in most cases) using a uniform maximum-effort cytoreductive algorithm throughout the study period. All surgeons were experienced in multivisceral procedures, including bowel and upper-abdominal surgery.^20^ Detailed operative techniques, including instruments, energy platforms, and perioperative pathways are provided in the supplementary method section and in the legend of the supplementary video.

Disease mapping included the peritoneal cancer index (PCI);^21,22^ surgical complexity was recorded intraoperatively. The default standardized sequence comprised infragastric omentectomy, pelvic peritonectomy, and targeted parietal peritoneal stripping. Upper-abdominal cytoreduction (right/left diaphragmatic peritonectomy with full-thickness resection when required, lesser-sac dissection, splenectomy ± distal pancreatectomy, hepatic surface stripping) was undertaken to achieve macroscopic clearance; full-thickness diaphragmatic defects were closed with nonabsorbable sutures and a drain when indicated (Medela AG, Baar, Switzerland).

Bowel surgery included low anterior resection, segmental colectomy, and small bowel resections including ileocecal resection when dictated by transmural involvement or unresectable serosal studding. Anastomoses were stapled or handsewn according to tissue quality; integrity was routinely leak-tested. A diverting stoma was created selectively on the basis of predefined criteria (e.g., low pelvic anastomosis, multiple anastomoses, compromised perfusion).

The surgical endpoint was complete macroscopic cytoreduction; any residual disease was measured in millimeters. Postoperatively, patients followed a protocolized Enhanced Recovery After Surgery (ERAS)-like pathway with early mobilization and feeding; drains were used selectively and removed early.

### Data Collection

Clinical data were extracted from electronic medical records, including demographics, comorbidities, American Society of Anesthesiologists (ASA) classification, and detailed surgical reports including 20–50 intraoperative photographs or videos per case. PCI was extracted from clinical records or retrospectively determined on the basis of surgical reports and photographs. The SCS^11^ was calculated to quantify procedural extent, including lymphadenectomy. According to Aletti et al. (2007),^11^ SCS ≥ 8 indicates high surgical complexity, while SCS of 4–7 indicates intermediate complexity. Postoperative complications during the first 30 days were graded using the Clavien–Dindo system.^23^ Follow-up was updated in December 2024 and used to calculate progression-free survival (PFS, time from diagnosis to last living information, recurrence, progression, or death) and overall survival (OS, time from diagnosis to last living information or death).

### Statistical Analysis

Binary logistic regression identified factors associated with major complications, defined as Clavien–Dindo grade ≥ IIIb within 30 days (grade IIIa requiring surgical, endoscopic, or radiological intervention without general anesthesia; grade IIIb requiring intervention under general anesthesia). Variables with significant correlation (*p* < 0.05) were further assessed in multivariate models to adjust for confounders. For evaluation of predictors for long-term outcome, univariate and multivariate Cox regression models were used. Median follow-up was estimated by the reverse Kaplan–Meier method; survival curves and medians were calculated using the Kaplan–Meier method.

## Results

**Fig. 1:**
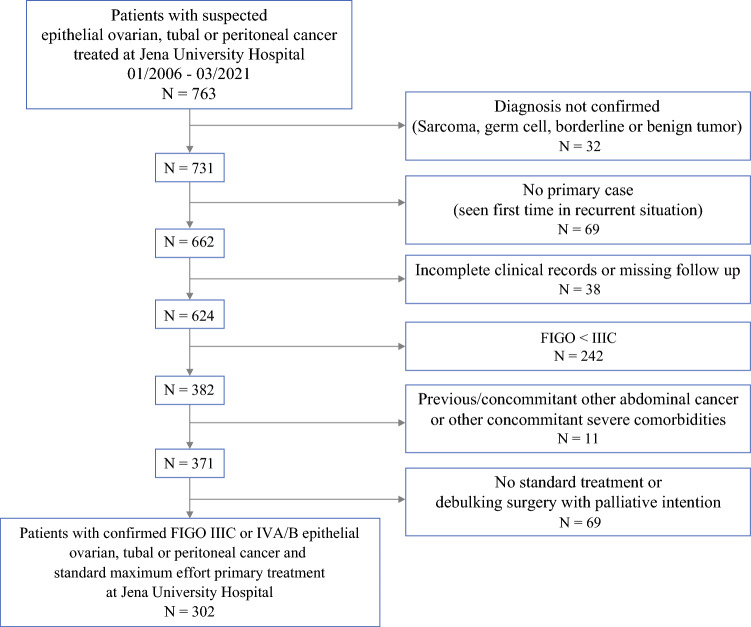
Flowchart: recruitment of study cohort; *FIGO* International Federation of Gynecology and Obstetrics ovarian cancer staging (version 2014)

In the study period (01/2006–03/2021), 763 patients were treated for suspected epithelial ovarian, tubal, or peritoneal cancer at our ESGO-certified center. After application of inclusion and exclusion criteria, the study cohort comprised 302 all-comers patients with FIGO (2014) stage IIIC or IVA/B epithelial ovarian, tubal, or primary peritoneal cancer who underwent maximum-effort cytoreductive surgery. All cases had complete perioperative and long-term follow-up data (Fig. 1).

### Baseline Characteristics and Standard Surgical Steps

Baseline characteristics and surgical complexity are presented in Table 1. Mean age at surgery was 63.3 years; more than 80% had high-grade serous tumors, and 77.2% were FIGO stage IIIC. PCI was found documented in the clinical records of 103 patients and was retrospectively determined for 104 patients (mean 19.4, SD 7.8). For 95 patients, PCI could not be determined.

**Table 1:** Baseline characteristics and surgical complexity in n = 302 patients with FIGO IIIC or IVA/B ovarian cancer treated with maximum effort cytoreductive surgery

Age [years], mean (range)		63.3 (26–88)
Comorbidities, *N* (%)	Hypertension	104 (34.4)
	Heart disease	37 (12.3)
	Diabetes	27 (8.9)
ASA, *N* (%)	1 or 2	138 (45.7)
	3 or 4	159 (52.6)
	Unknown	5 (1.7)
FIGO, *N* (%)	IIIC	233 (77.2)
	IVA	33 (10.9)
	IVB	36 (11.9)
Histology, *N* (%)	Serous	258 (85.4)
	Mucinous	14 (4.6)
	Endometrioid	12 (4.0)
	Other	18 (6.0)
Grading, *N* (%)	High	273 (90.4)
	Low	4 (1.3)
	Unknown	25 (8.3)
Neoadjuvant chemotherapy *N* (%)		42 (13.9)
Complete cytoreductive surgery, *N* (%)		259 (85.8)
Surgical complexity score (SCS), Mean (SD)		9.7 (3.0)
SCS group, *N* (%)	Intermediate (4–7)	81 (26.8)
	High (8–16)	221 (73.2)
Surgical intervention, *N* (%)	Omentectomy	299 (99.0)
	Pelvic peritoneum stripping	291 (96.4)
	TH-BSO	283 (93.7)
	Pelvic lymphadenectomy	246 (81.5)
	Paraaortic lymphadenectomy	245 (81.1)
	Diaphragm stripping/resection	223 (73.8)
	Abdominal peritoneum stripping	192 (63.6)
	Rectosigmoidectomy/T-T-anastomosis	175 (57.9)
	Large bowel resection/s	120 (39.7)
	Small bowel resection/s	77 (25.5)
	Liver resection/s	25 (8.3)
	Splenectomy	21 (7.0)
Stoma, *N* (%)	Ileostoma	16 (5.3)
	Colostoma	50 (16.6)
Combinations of bowel resections, *N* (%)	No bowel resections	86 (28.5)
	Rectosigmoidectomy/T-T-anastomosis only	82 (27.2)
	Large bowel resection only	17 (5.6)
	Small bowel resection only	4 (1.3)
	Small bowel + large bowel resection	20 (6.6)
	Rectosigmoidectomy + large bowel resection	40 (13.2)
	Rectosigmoidectomy + small bowel resection	10 (3.3)
	Rectosigmoidectomy + large bowel + small bowel	43 (14.2)

Surgical complexity score (SCS) according to Aletti et al.^11^

*ASA* American Society of Anesthesiologists physical status classification, *FIGO* International Federation of Gynecology and Obstetrics ovarian cancer staging (version 2014), *TH-BSO* total hysterectomy with bilateral salpingo-oophorectomy

Neoadjuvant chemotherapy was administered in 13.9% (*n* = 42). A total of 40 patients received carboplatin plus paclitaxel, one received carboplatin plus gemcitabine, and one received carboplatin monotherapy. Most patients received three (*n* = 26) or six (*n* = 12) cycles; one (*n* = 1), four (*n* = 2), or five (*n* = 1) cycles were given exceptionally. Six patients received bevacizumab in addition; bevacizumab was withheld for at least 6–8 weeks before surgery to reduce the risk of wound-healing complications. Common comorbidities included hypertension (34.4%), heart disease (12.3%), and diabetes (8.9%). Standard procedures included TH-BSO (93.7%), infragastric omentectomy (99.0%), pelvic peritoneum stripping (96.4%), and systematic pelvic and paraaortic lymphadenectomy (81%), reflecting routine care outside the LION^24^ trial context or its adoption after the publication.

Time to chemotherapy (TTC) was available for 176/302 patients (58.3%); the mean interval from surgery to chemotherapy was 49.6 days (SD 28.3). Chemotherapy was frequently initiated at referring outpatient centers, accounting for incomplete institutional capture of TTC.

### Extent of Organ Resections and Surgical Complexity

To achieve complete cytoreduction or restore functional organ anatomy, additional resections included diaphragm stripping/resection (73.8%), abdominal peritoneum stripping (63.6%), rectosigmoidectomy with T-T anastomosis (57.9%), large bowel segments (39.7%), small bowel segments (25.5%), liver segments (8.3%), and spleen (7.0%) (Table 1).

Among 216 patients with bowel resections, 47.7% (*n* = 103) had a single-site resection, mostly rectosigmoid (*n* = 82). Combined resections involved rectosigmoid and colon segments (*n* = 40), rectosigmoid and small bowel segments (*n* = 10), or all three regions (*n* = 43). In 20 cases, rectosigmoid was preserved despite other bowel segment resections. The ileocecal region was resected in 24.5% of bowel resections (*n* = 53). These cases were classified as combined small and large bowel resections and are therefore counted in both the small bowel and large bowel resection categories in Table 1 (overlap; categories are not mutually exclusive). Mean length of resected small bowel was 20.6 cm (max 75 cm), with tumor infiltration confirmed in 87% of specimens. Ileostomies were performed in 5.3% of patients, colostomies in 16.6%.

Complete tumor resection was achieved in 85.8% of patients but was not feasible in our hands in 14.2% of patients due to tumor infiltration of inaccessible or vital structures. The mean SCS was 9.7, with 73.2% classified as high complexity (SCS 8–16).

Lymphadenectomy involved systematic dissection up to the renal veins and selective removal of suspicious nodes above. The number of resected lymph nodes ranged up to 207 (mean 83; median 79). Lymph node metastases were histologically confirmed in 148/249 (59.4%) of cases (52 missing, 49 no metastases).

### Risk Factors for Major Postoperative Complications

Postoperative complications (30-day) were graded using the Clavien–Dindo system (detailed grading listed in Supplementary Table S1). A total of 64 (21.2%) patients had no postoperative complications. Minor complications (grade I–IIIa) occurred in 51.0% of patients, major complications (grade IIIb–IVb) in 21.2%. A total of 17 patients (5.6%) died; causes included cardiovascular/pulmonary failure (*n* = 6), multiorgan failure (*n* = 5), and peritonitis/sepsis (*n* = 4). In two cases, cause of death was not documented. Postoperative course was undocumented in three cases (1%).

Mean hospital stay was 15–19 days in patients with no or minor postoperative complications but was prolonged in case of major complications (up to 37 days). Reoperation was necessary in 59 patients (19.5%). Most frequently, re-laparotomy had to be performed (12.3%), and in some cases surgical therapy of disturbed wound healing (2.3%) and pleural puncture (2.0%) was necessary (Supplementary Table S1). Anastomotic leakage occurred in 13 of 216 patients with bowel resections (6.0%), with no significant association to transitory stoma formation (*p* = 0.759; Supplementary Table S2).

Excluding patients who received NACT, the surgery-to-chemotherapy interval (TTC) differed by postoperative complication severity (Kruskal–Wallis *p* < 0.001): major complications (Clavien–Dindo ≥ IIIb) 66.4 ± 37.1 days, minor complications (I–IIIa) 45.7 ± 24.1 days, and no complications 45.8 ± 29.2 days.

Binary logistic regression identified risk factors for major complications (Clavien–Dindo ≥ IIIb): high surgical complexity, specifically large bowel segment resection, splenectomy, abdominal peritoneum stripping, colostomy, and small bowel segment resection (Supplementary Table S3). Age or comorbidities showed no association with postoperative morbidity.

**Fig. 2:**
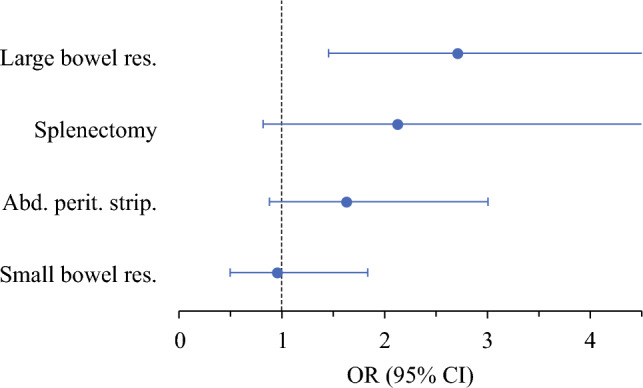
Predictive factors for 30-day postoperative major complications (≥ IIIb according to Clavien–Dindo) after maximum effort debulking surgery in patients with FIGO IIIC or IVA/B ovarian cancer (*n* = 299) analyzed by multivariate logistic regression; only surgical steps were included, which showed significant results in univariate analysis (Supplementary Table S3); for three patients of the study cohort (*n* = 302), data on postoperative course were not available

In multivariate binary logistic regression analysis, only large bowel resection remained an independent impact factor on postoperative course (OR 2.708, 95% CI 1.449–5.061, Supplementary Table S3 and Fig. 2), while other bowel surgery (small bowel resection or rectosigmoidectomy) did not significantly impact complication rates.

### Factors Impacting on Long-Term Outcome

To reduce bias from early postoperative events, only patients with > 3 months of follow-up (*n* = 263) were included in long-term outcome analyses. Median follow-up was 73.7 months. In univariate Cox regression, complete macroscopic resection significantly improved PFS and OS, while age, small bowel resection, large bowel resection, and abdominal peritoneum stripping had a negative effect (Supplementary Table S4). General high surgical complexity (SCS ≥ 8) and neoadjuvant chemotherapy had no significant effect on long-term outcome; this was also the case for systematic lymphadenectomy and time to chemotherapy. However, analysis on time to chemotherapy is not reliable because of many missing values (73/225, 32.4%). Furthermore, survival outcome was not dependent on the number of resected lymph nodes nor the presence of lymph node metastases.

**Fig. 3:**
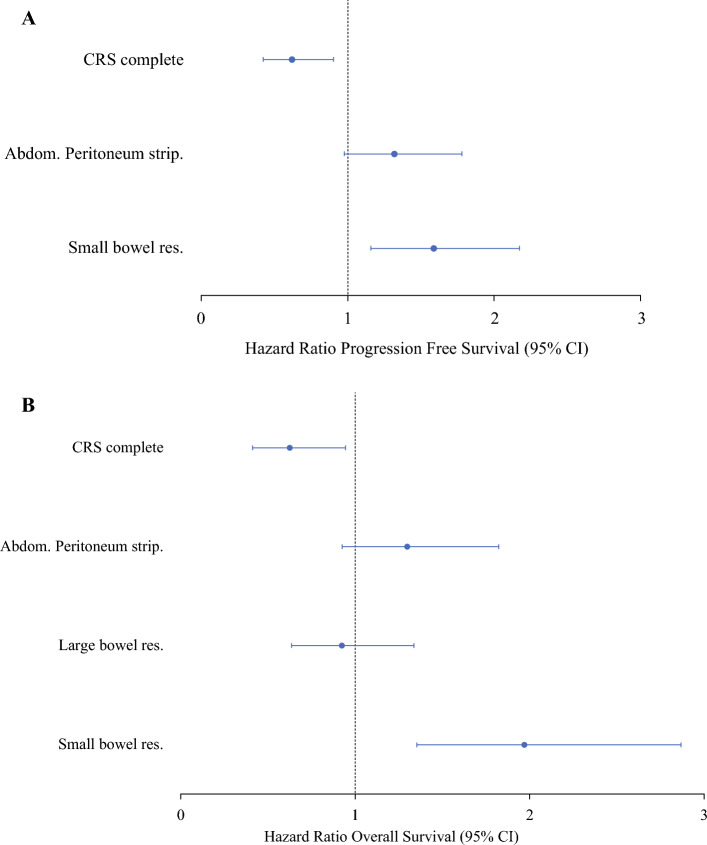
Factors impacting on survival in patients with FIGO IIIC or IVA/B ovarian cancer after maximum effort debulking surgery (*n* = 263), analyzed by multivariate Cox regression, adjusted for age; patients were included if follow-up beyond 3 months was available; only factors that showed significant results in univariate analysis were included (Supplementary Table S4); A Progression-free survival (PFS); **B** Overall survival (OS)

In multivariate analysis adjusted for age, only small bowel resection remained an independent negative prognostic factor (PFS: HR 1.586, 95% CI 1.158–2.17; OS: HR 1.971, 95% CI 1.355–2.867; Fig. 3).

### Survival Analysis Based on Small Bowel Resection

**Fig. 4:**
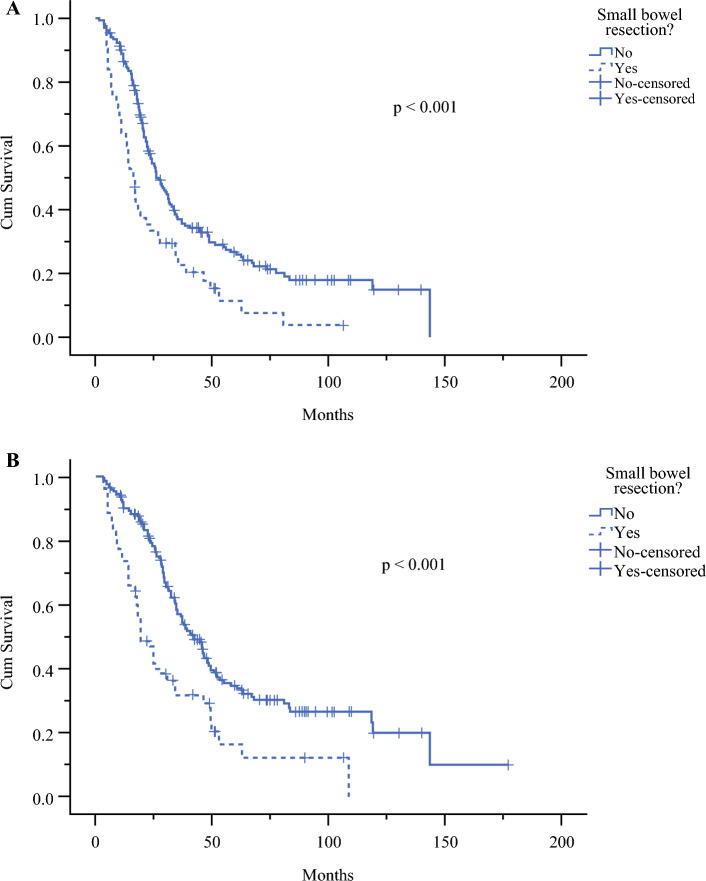
Kaplan–Meier curve depending on necessity for small bowel resection/s during maximum effort debulking surgery; patients were included if they had FIGO IIIC or IVA/B ovarian cancer, complete tumor resection, and follow-up beyond 3 months (*n* = 225); *p*-value according to log rank test; **A** Progression-free survival (PFS); **B** Overall survival (OS)

Subgroup analysis (Fig. 4 and Supplementary Table S5) showed that patients who underwent complete CRS without small bowel resection had significantly (*p* < 0.001) increased long-term survival, corresponding to a 10-month longer median PFS and a 22.8-month longer median OS. The 3-year overall survival rate was 57% without versus 31.8% with small bowel resection.

### Detailed Analysis of the Combined Bowel Resections

**Fig. 5:**
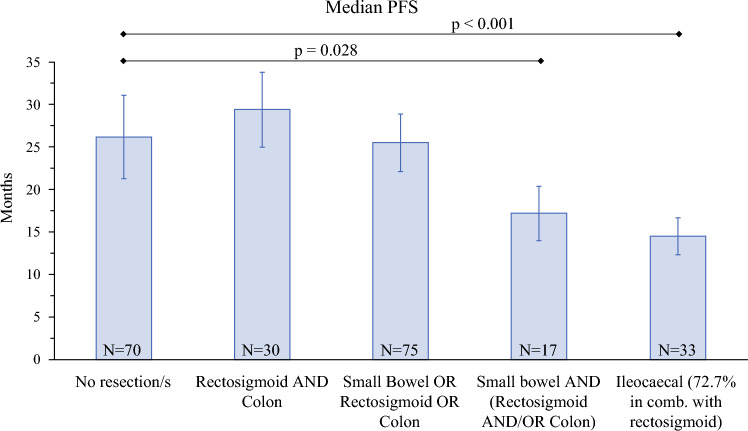
Median progression-free survival (PFS, months) depending on type and combination of bowel resections during maximum effort debulking surgery, calculated with the Kaplan–Meier method; patients were included (*n* = 225) if they had FIGO IIIC or IVA/B ovarian cancer, complete tumor resection, and follow-up beyond 3 months; *p*-values according to log rank test

Because the patients analyzed above comprise patients with resection/s exclusively in the region of the small intestine as well as patients with small bowel resection/s in combination with resection/s in other bowel regions, we further analyzed combinations of bowel resections in relation to long-term outcomes post-complete CRS (Fig. 5, Supplementary Table S6, and Supplementary Figs. S7, S8).

Patients with a single bowel resection region (rectosigmoid OR colon OR small bowel) had survival comparable to patients without bowel resection (months: PFS: 25.5 ± 3.4 versus 26.2 ± 4.9,* p* = 0.448; OS: 38.7 ± 5.0 versus 46.0 ± 5.7, *p* = 0.815). This was also observed for patients with rectosigmoid in combination with colon resections (months: PFS 29.4 ± 4.4, *p* = 0.515; OS: 49.3 ± 10.7, *p* = 0.575).

Because ileocecal resection inherently represents combined small and large bowel resection, it was analyzed as a separate subgroup. Among patients without ileocecal resection, combined small bowel plus large bowel and/or rectosigmoid resections were associated with worse outcomes (PFS 17.2 ± 3.2 months, *p* = 0.028; OS 24.6 ± 5.7 months, *p* = 0.062). Outcomes were poorest in patients undergoing ileocecal resection (PFS 14.5 ± 2.2 months, *p* = 0.001; OS 19.2 ± 3.6 months,* p* < 0.001).

Of note, the total peritoneal carcinoma index (PCI) and the PCI for the regions of the small intestine (regions 9–12) did not show a significant difference between patients with ileocecal resection compared with patients with other bowel resections, such as rectosigmoid and colon (mean ± SD: total PCI 20.4 ± 6.2 versus 18.8 ± 4.9,* p* = 0.533; PCI_9–12 6.3 ± 3.3 versus 4.5 ± 3.1, *p* = 0.076) or single region bowel resections (total PCI, *p* = 0.487; PCI_9–12, *p* = 0.218) (Supplementary Tables S9 and S10).

Furthermore, overall surgical complexity, assessed by the SCS, was not different between patients with ileocecal resection and patients with only rectosigmoid and colon resections (mean ± SD, 12.6 ± 2.1 versus 12.7 ± 1.5, *p* = 0.922, Supplementary Table S11). However, compared with patients with single bowel resections (10.3 ± 1.6), surgical complexity was significantly higher in patients with ileocecal resection (*p* < 0.001), probably because in 72.7% of cases, the rectosigmoid also had to be removed.

## Discussion

In this 15-year real-world cohort from a certified high-volume center, we analyzed surgical complexity, perioperative morbidity, and oncologic outcomes in patients with stage IIIC–IV epithelial tubo-ovarian/primary peritoneal cancer, managed with a uniform maximum-effort cytoreductive strategy throughout the study period.

### Brief Presentation of Main Results

Complete macroscopic cytoreduction was achieved in 85.8% under a standardized algorithm. Site-specific bowel involvement (small bowel, and independently, ileocecal) was associated with inferior PFS and OS despite complete cytoreduction, adding prognostic information beyond overall surgical complexity. Large-bowel resection independently increased major postoperative morbidity. Our data support the search for a more stratified treatment planning, which in the future could be supported by artificial intelligence (AI).^25,26^ With the limited sensitivity of CT for small bowel serosal disease, resectability assessment often required additional workup such as diagnostic laparoscopy in selected cases. Patients receiving NACT followed by complete cytoreduction had survival comparable to primary surgery, supporting selective use of neoadjuvant strategies in complex tumor spread, including small bowel or ileocecal involvement, and in frail or medically complex patients.

### Results in the Context of Literature

Advanced ovarian cancer frequently presents with diffuse peritoneal carcinosis involving the intestinal tract. As complete macroscopic tumor removal remains the strongest prognostic factor,^6–8^ guidelines endorse primary maximum-effort cytoreduction. This includes tumor stripping and/or resection of extra-pelvic structures, especially small and large bowel, diaphragm, abdominal peritoneum, liver, and spleen, whenever required to achieve complete resection.^11^ Systematic pelvic and paraaortic lymphadenectomy was routinely performed at our institution until the LION trial,^24^ except for cases recruited in the experimental arm of this trial.

Achieving complete cytoreduction requires specialized expertise and coordinated perioperative care but carries substantial risk. In the cohort presented in this study, bowel resections were pivotal for both morbidity and survival. While large bowel resections increased postoperative complications, small bowel resections independently predicted worse long-term outcomes, likely reflecting not only surgical burden, but also particularly aggressive tumor spread. Ileocecal resections, required in nearly a quarter of cases, were linked to the poorest survival. On the basis of our data, this was not explainable by differences in extent of peritoneal carcinosis or tumor spread to small bowel or overall surgical complexity. This region’s dense lymphovascular network and immune tissue [e.g., Peyer’s patches, gut-associated lymphoid tissue (GALT)] may promote tumor progression and immune evasion. Because of its vital role in vitamin B12 and bile salt absorption, microbiota regulation, and intestinal transit control via the ileocecal valve, removal of the ileocecal region could compromise nutritional status and tolerance to systemic therapy. To our knowledge, this is the first study to demonstrate that ileocecal involvement independently predicts poor survival despite complete cytoreduction in a homogeneously treated PDS cohort. In contrast to the study of Bisgin et al.,^27^ where ileal resection was not an independent prognostic factor after adjustment for tumor burden and surgical complexity, our findings emphasize the importance of preserving the ileocecal region whenever feasible in cytoreductive surgery, and support its inclusion in future stratification models. Our findings align with GOG 182, including 2655 patients with ovarian or primary peritoneal cancer showing that baseline disease burden remains prognostically relevant even after complete gross resection.^8,28^

Bowel resections increase postoperative risk, primarily due to anastomotic leakage (AL), reported in 2.9–7.1% of patients,^29,30–36^ potentially leading to sepsis, reoperation, prolonged hospitalization, and delayed chemotherapy.^20,37^ Although transitory stomas are often used to mitigate this risk and support recovery, we observed no significant association between stoma formation and AL. Recent studies likewise found no consistent protective effect,^34–36^ but point to other factors for AL risk, such as malnutrition. To our knowledge, this is the first study addressing this question in a homogeneously treated PDS cohort with > 80% macroscopic complete resection. Future subgroup analyses in large prospective trials, such as TRUST,^38^ should further explore the prognostic impact of bowel surgery on both short- and long-term outcomes.

While the LION^24^ trial found no survival benefit from systematic lymphadenectomy in clinically node-negative patients, it did not assess subgroup effects of occult nodal metastases. A Surveillance, Epidemiology, and End Results (SEER)-based analysis^39^ reported improved outcomes with > 10 nodes removed, though extent of dissection and nodal status were unspecified. In our cohort, systematic lymphadenectomy or the number of removed lymph nodes (up to 207 nodes resected, supplementary video) had no impact on survival. Of systematically node-dissected patients, 59.4% had histologically confirmed lymph node metastasis, which showed no significant correlation with long-term outcome. These data suggest that occult nodal metastasis add limited prognostic value for long-term survival in FIGO IIIC/IV disease after complete cytoreduction.

Splenectomy frequency was low in our cohort (7.0%), reflecting a selective, organ-preserving upper-abdominal approach. Capsular or hilar involvement was managed by partial capsulectomy and meticulous hilar skeletonization with topical hemostatics, reserving splenectomy for parenchymal infiltration or uncontrolled bleeding. Recent series likewise suggest that splenectomy primarily carries higher postoperative morbidity rather than conferring an intrinsic survival advantage. A spleen-preserving approach may lower perioperative morbidity (pancreatic fistula, subphrenic abscess, hemorrhage) and preserve splenic immune function, consistent with recent meta-analyses.^40^

Our ≥ IIIb major-complication rate was 21.2% and sits within prospective benchmarks. The prospective RISC-GYN cohort reported 63.2% complete cytoreduction and 17.9% severe complications (≥ IIIb).^41^ In a Finnish real-world PDS series, complete macroscopic cytoreduction was achieved in 53.8% after extensive surgery, while Clavien–Dindo grade 3+ complications occurred in 41.2%.^42^

The 30-day mortality was 5.6% in this all-comers stage IIIC–IV cohort treated with a maximum effort strategy. A systematic review reported average postoperative mortality of 3.7% across population-based series, while earlier high-complexity tertiary-center experiences in advanced disease document rates around 6% in highly selected high-risk referrals.^43^ Population-level data likewise show a 90-day mortality of 7.2% after primary cytoreduction.^44^ These benchmarks contextualize our program-level outcome and underscore that all-comers cohorts, unlike trial-selected populations with stringent eligibility and lower comorbidity burden, carry intrinsically higher perioperative risk, reinforcing the need for risk-adapted MDT triage, selective use of NACT followed by IDS, and ERAS-aligned perioperative care, which can be safeguarded only in high-volume, specialized centers.

Randomized trials have shown NACT followed by interval debulking surgery (IDS) to be noninferior to primary surgery (PDS) in selected patients, although surgical quality varied across studies (CHORUS, EORTC 55971, III SCORPION-NCT01461850).^12,13,15^ The TRUST study, led by AGO-OVAR as presented at American Society of Clinical Oncology (ASCO) 2025,^45^ was an international, randomized, multicenter phase III trial investigating the optimal timing for surgical intervention, comparing PDS with IDS following three cycles of NACT. The primary endpoint, OS, was not significantly improved with PDS (median 54.3 versus 48.3 months; HR 0.89; *p* = 0.24). By contrast, PFS was significantly longer with PDS (median 22.1 versus 19.7 months; HR 0.80; *p* = 0.018). Surgical morbidity was 18% after PDS and 12% after IDS.

### Strengths and Limitations

For some subgroup analyses, this retrospective single-center study may be underpowered, e.g., regarding impact of different combinations of bowel resections on long-term survival. Furthermore, exhaustive analysis of the impact of postoperative complications on time to chemotherapy and the effect of delayed chemotherapy on long-term outcome was not possible because of missing data due to a trend to outpatient chemotherapy. Given our referral model, the observed TTC (mean 49.6 days) reflects case mix and cross-institutional coordination rather than systematic in-hospital delay; although ≤ 42 days is commonly targeted, literature on TTC and outcomes is mixed, with some benefit signals for ≤ 28 days in advanced-stage or IDS cohorts. However, the presented study offers robust real-world data from an all-comers cohort treated over 15 years at a certified high-volume center. Unlike RCTs, it reflects the consistent application of maximum-effort cytoreductive surgery in clinical routine, offering a comprehensive evaluation of a widely implemented surgical strategy across specialized centers. Given the 2006–2021 study period, evolving systemic standards, including the staged introduction of poly (ADP-ribose) polymerases (PARP) inhibitor maintenance, may have influenced survival and introduced era-related confounding that cannot be fully addressed in this retrospective design. EU approvals began with olaparib in 2014 for a limited BRCA mutated, platinum-sensitive relapsed subgroup, expanded with niraparib in 2017 for recurrent disease regardless of BRCA status, and extended to selected first-line maintenance indications from 2019, however, these indications applied to relatively small subsets within this all-comers cohort.

### Implications and Conclusions

Small bowel resection, and independently, ileocecal involvement, were associated with inferior long-term outcome despite complete cytoreduction. Together with comparable PCI and surgical complexity across subgroups, this defines a high-risk phenotype that should guide preoperative counseling and patient selection. In practice, patients with extensive small bowel or ileocecal disease, particularly if frail or medically complex, may be better served by primary systemic therapy with planned interval cytoreduction. Preoperative stratification should combine contrast-enhanced cross-sectional imaging with dedicated small bowel assessment, multidisciplinary tumor board review, and when resectability remains uncertain, selective diagnostic laparoscopy. AI-assisted image analysis is a promising adjunct but warrants prospective validation. Large-bowel resection was independently associated with major morbidity, while temporary stoma formation was not associated with reduced leakage risk. These findings support individualized planning within prehabilitation and ERAS-like frameworks and justify prospective multicenter validation to refine risk-adapted pathways.

## Supplementary Information

Below is the link to the electronic supplementary material.

Supplementary file1 (DOCX 15 kb)

Supplementary file2 (DOCX 112 kb)

Supplementary file3

## Data Availability

Data are available from the authors upon reasonable request.
