# Supplementary material for: Ileocecal and Small Bowel Involvement Are Independently Associated with Inferior Survival Despite Complete Cytoreduction in FIGO IIIC–IV Tubo-Ovarian and Primary Peritoneal Carcinoma
Source: Ann Surg Oncol. 2026 Apr 12;33(7):6639–51. doi: 10.1245/s10434-026-19485-5 (PMC13242446; doi:10.1245/s10434-026-19485-5)
Supplement: Supplementary file 1 — Supplementary file1 (DOCX 15 kb) [file 10434_2026_19485_MOESM1_ESM.docx]

## Supplementary Video S1 Legend:

Final Situs After Complete Cytoreduction in Advanced FIGO IIIC High-Grade Serous Tubo-Ovarian Carcinoma

Duration: 4:44 minutes | Recording device: HD laparoscope, Olympus® | Frame rate: 480 bps

Intraoperative final inspection documenting the operative field after complete macroscopic cytoreduction in a patient with FIGO stage IIIC epithelial tubo-ovarian cancer with diffuse peritoneal dissemination, diaphragmatic involvement, and bulky pelvic and para-aortic nodal disease extending above the left renal vein. The video is illustrative of the documentation protocol, it does not imply routine, template-based pelvic and para-aortic lymphadenectomy or total parietal peritonectomy in all patients; in our practice these procedures are performed selectively for clinically or radiologically suspicious disease, consistent with current evidence.

The video begins with a panoramic view of the pelvis seen from below, demonstrating complete peritoneal stripping, deperitonealization of the bladder dome, and resection of the rectosigmoid colon prior to anastomosis. Bilateral iliac vessels (common and external arteries and veins) are exposed. The hypogastric plexus and periaortic nerve-bearing tissue are preserved. The pelvic cavity contains irrigation fluid (Aqua destillata). Both ureters are looped with yellow vessel loops solely for identification to preserve vascular integrity.

The dissection proceeds cranially, showing exposed iliopsoas muscles and complete removal of paracaval and para-aortic lymphatic tissue. Key structures including the superior mesenteric artery and the left renal vein crossing anterior to the aorta are visualized. Titanium clips are placed on major venous and on lymphatic tributaries to prevent lymphatic leakage.

A suprarenal lymphadenectomy necessary due to gross nodal involvement is shown. Subsequent scenes demonstrate lymphadenectomy at the hepatic hilum and clearance posterior to the caudate lobe. The falciform ligament has been dissected using electrocautery; the liver is fully mobilized. On the right diaphragm, a full-thickness resection with partial pleurectomy was performed due to tumor involvement. A laparoscopic view confirms no intrapleural disease.

The fourth minute inspects the spleen and left upper quadrant. The left diaphragm is also fully stripped of peritoneum. A protective surgical towel is placed over the spleen to prevent capsular injury. The splenic hilum appears intact. A complete omentectomy has been performed, and splenic vasculature is preserved. The left kidney and surrounding perinephric fat are shown unaltered.

In the final minute, the pelvis is re-examined. The mobilized colon is positioned tension-free for colorectal anastomosis. Bilaterally looped ureters are visible. Vaginal cuff sutures aid exposure. The deperitonealized pelvis is shown in full. Gentle retraction highlights anatomical relationships between bladder, vagina, and resection margins. A radical hysterectomy has been performed with autonomic nerve preservation.

Summary: This unedited surgical video illustrates the situs after high-complexity cytoreductive procedure including total abdominal peritonectomy, suprarenal and infrarenal lymphadenectomy (including hepatic hilum), anterior rectal resection, diaphragmatic stripping and resection, and complete omentectomy—resulting in a macroscopically tumor-free situs.
