# Supplementary material for: Ileocecal and Small Bowel Involvement Are Independently Associated with Inferior Survival Despite Complete Cytoreduction in FIGO IIIC–IV Tubo-Ovarian and Primary Peritoneal Carcinoma
Source: Ann Surg Oncol. 2026 Apr 12;33(7):6639–51. doi: 10.1245/s10434-026-19485-5 (PMC13242446; doi:10.1245/s10434-026-19485-5)
Supplement: Supplementary file 2 — Supplementary file2 (DOCX 112 kb) [file 10434_2026_19485_MOESM2_ESM.docx]

**Supplementary text and Tables/Figures S1 through S11**

**Manuscript:** Ileocecal and Small-Bowel Involvement Are Independently Associated with Inferior Survival Despite Complete Cytoreduction in FIGO IIIC–IV Tubo-Ovarian and Primary Peritoneal Carcinoma

**Authors:** Ingo B. Runnebaum, Angela Kather, Clara Evangelia Goerdt, Huyen Thi Thanh Nguyen, Davit Bokhua

**Supplementary method section:**

All surgeries were performed by at least one of three board-certified gynaecologic oncologists, with IBR serving as primary surgeon in most cases. All surgeons were experienced in multivisceral procedures including bowel and upper-abdominal surgery and followed a standardized protocol aiming for complete macroscopic tumour resection. The same treatment strategy was consistently followed during the whole study period. Dissection was performed with monopolar diathermy with TriVerse™ in combination with the ForceTriad™ energy platform and LigaSure™ (Medtronic, Minneapolis, MN, USA) or MarClamp® (KLS Martin Group, Germany) for vessel sealing, and an ultrasonic dissector as appropriate (Harmonic™, Ethicon, Cincinnati, OH, USA). minimizing blood loss. Smoke evacuation and sealing techniques were employed in all cases. An intensive care unit (ICU) bed was pre-reserved for all patients undergoing this level of surgical complexity. Patients were positioned in the supine lithotomy position using foam-padded leg supports in a slightly flexed posture. Arms were abducted at less than 90 degrees and carefully padded to prevent brachial plexus injury. Sequential compression devices (Kendall SCD™ 700 Series, Medtronic, Minneapolis, MN, USA; formerly Covidien) were applied bilaterally to reduce the risk of venous thromboembolism. Preoperative skin antisepsis was applied, followed by sterile single-use draping.

After midline laparotomy from xiphoid to pubis, wound infiltration with ropivacaine was applied to reduce postoperative pain 22. A self-retaining retractor system (Sattler frame, Thüringen, Germany) was mounted. Intraoperative active warming was maintained using forced air warming blankets, covering the upper body and extremities throughout the procedure. Transfusions with fresh frozen plasma or packed red blood cells were administered in coordination with anesthesia. The greater omentum was resected en bloc with preservation of gastroepiploic vessels and vagal innervation. The omentum was dissected from the transverse colon, adhesions lysed, and serosal defects repaired. Gastric infiltration required full-thickness resection and primary closure. The peritoneal cavity was inspected to assess spread and plan resection. Superficial liver metastases were removed via monopolar coagulation; suspicious nodes at the hepatoduodenal ligament and celiac trunk were resected. Right diaphragmatic stripping was followed by laparoscopic inspection of the pleural space if invaded; full-thickness defects were closed with non-absorbable sutures and drains placed (Sahara®, Medela AG, Switzerland). On the left, stripping was completed and retrocrural nodes palpated, suspicious retroperitoneal and interaortocaval nodes were resected with vascular preservation. Peritoneal implants on mesocolon and mesentery were sharply excised; paracolic gutter involvement led to peritoneal resection. Mesenteric lymphadenopathy was dissected with vessel preservation.

Lymphadenectomy and parietal peritonectomy approach. Routine template pelvic and para-aortic lymphadenectomy was not performed following the LION study results. Systematic lymphadenectomy was selectively undertaken for bulky or clinically/radiologically suspicious nodes following multidisciplinary tumor board (MDT) review. Extensions of para-aortic dissection cranial to the left renal vein followed the same selective policy. Total parietal peritonectomy was reserved for diffuse parietal peritoneal involvement when limited stripping would be incomplete. This policy is consistent with contemporary evidence, including the AGO-OVAR LION randomized trial, which demonstrated no progression-free or overall survival benefit but higher morbidity for routine systematic lymphadenectomy in patients with complete intra-abdominal macroscopic cytoreduction and clinically normal nodes.

In the lower abdomen and pelvis, small-bowel loops were carefully packed away using warm, moist pads. Ureters were bilaterally identified and freed together with their vasculature to avoid injury during subsequent resection steps, and autonomic pelvic nerve structures were preserved wherever feasible. In cases of pelvic conglomerate tumours, complete peritonectomy was performed, including stripping from the pelvic sidewalls, bladder peritoneum, and posterior cul-de-sac. When resection of the upper or mid rectum was required, a nerve-sparing en bloc pelvic resection was carried out using a modified Hudson technique. The uterus and rectum were resected en bloc by serial clamping and division using curved Wertheim clamps. The rectum was transected using a curved linear stapler (e.g., Medtronic Covidien™), and the specimen (envelope resection) was delivered intact. Intraoperative lavage was continuously performed with warm sterile water (37°C) to reduce tumour cell dissemination. Reconstruction was completed with a stapled colorectal anastomosis using a circular end-to-end anastomosis device (e.g., Covidien™ DST Series™ EEA™, Medtronic/Covidien, Minneapolis, MN, USA), inserted transanally. The anvil was secured proximally, and a single-use protective washer (tilting anvil cap) was applied to facilitate correct alignment and ensure full-thickness tissue approximation. Air leak testing was routinely performed to verify anastomotic integrity. Whenever feasible, a diverting transitory protective stoma was avoided, provided anastomotic perfusion and integrity were judged optimal intraoperatively.

Completeness of cytoreduction was assessed intraoperatively by the surgical team, including the senior gynecologic oncologist (mostly IBR). Complete resection was defined as no visible or palpable tumour in abdominal, pelvic, intraperitoneal, or extraperitoneal compartments. Final inspection included systematic exposure, palpation, and laparoscopic documentation of all peritoneal and retroperitoneal surfaces (see Supplementary Video S1). Findings were recorded immediately postoperatively. Surgical complexity was not limited by time or institutional constraints.

A restrictive stoma policy was consistently applied. Nerve-sparing techniques were prioritized, including meticulous dissection of the gastrohepatic ligament with preservation of vagal branches and gastric vasculature to prevent gastroparesis and ischemia. When oncologically safe, rectosigmoid-sparing en bloc pelvic resections (“shaving”) were performed to avoid unnecessary bowel resection and maintain continuity. During rectosigmoid resections using the modified Hudson technique, mesorectal preservation and protection of the hypogastric nerves and superior hypogastric plexus were emphasized to reduce autonomic nerve injury. This function-preserving approach aimed to maintain postoperative urinary, bowel, and sexual function in patients undergoing maximum-effort surgery.

All patients underwent an individualized prehabilitation protocol aligned with Enhanced Recovery After Surgery (ERAS) principles, including interdisciplinary anesthesiology evaluation, correction of electrolyte imbalances, hemoglobin and albumin optimization, and encouragement of moderate physical activity. Nutritional supplementation was prescribed as needed. Continuous psychological support was provided pre- and postoperatively. Intraoperative optimization included transfusion with blood products or electrolytes as indicated. Postoperatively, patients were initially transferred intubated to the ICU for monitoring and extubation. Early physiotherapy and oral nutrition were initiated. Surgical drains were placed selectively and removed early according to a restrictive management protocol.

Supplementary Table S1: Details of postoperative course.

| **Clavien-Dindo** | | **N (%)** | **Hospital stay [days], mean (SD)** | **Re-Interventions** | |
| --- | --- | --- | --- | --- | --- |
|  |  |  |  | Intervention | N |
| No postoperative complications | | 64 (21.2) | 17.2 (7.4), missing: 3 | - | |
| I: deviation from the normal postoperative course without need for treatment (except: antiemetics, antipyretics, analgetics, diuretics, electrolytes,  physiotherapy, infections opened at the bedside) | | 17 (5.6) | 15.1 (5.5), missing: 1 | - | |
| II: pharmacological treatment with drugs other than such allowed for grade I complications, blood transfusions and total parenteral nutrition | | 105 (34.8) | 17.6 (6.4), missing: 1 | - | |
| III: Requiring surgical, endoscopic or radiological intervention | IIIa: intervention not under general anesthesia | 32 (10.6) | 19.2 (7.0) | - | |
|  | IIIb: intervention under general anesthesia | 33 (10.9) | 34.7 (16.3), missing: 4 | Re-laparotomy | 21 |
|  |  |  |  | Surgical therapy of disturbed wound healing | 7 |
|  |  |  |  | Other | 5 |
| IV: Life-threatening complication requiring IC/ICU-management | | 2 (0.7) | 21.0 (29.7) | Re-laparotomy | 1 |
| IVa: single organ dysfunction (including dialysis) | | 25 (8.3) | 31.4 (20.7) | Re-laparotomy | 7 |
|  |  |  |  | Pleural puncture | 3 |
|  |  |  |  | Other | 2 |
| IVb: multi organ dysfunction | | 4 (1.3) | 37.0 (5.3) | Re-laparotomy | 2 |
| V: Death of a patient | | 17 (5.6) | 13.4 (6.8) | Re-laparotomy | 6 |
|  |  |  |  | Pleural puncture | 3 |
|  |  |  |  | Other | 2 |
| Unknown postoperative course | | 3 (1.0) | - | - | |

**Supplementary Table S2:** Anastomosis leakage and stoma formation (ileostoma n=16, colostoma n=50) in n=216 patients with FIGO IIIC or IVA/B ovarian cancer after maximum effort cytoreductive surgery with bowel resections. Fisher’s Exact Test.

|  | | Stoma | | *p*-value |
| --- | --- | --- | --- | --- |
|  |  | No | Yes |  |
| Anastomosis leakage, n (%) | No | 140 (93.3) | 63 (95.5) | 0.759 |
|  | Yes | 10 (6.7) | 3 (4.5) |  |

**Supplementary Table S3:** Binary logistic regression for risk of major complications (Clavien-Dindo ≥ IIIb), n=299 (three patients with unknown postoperative course excluded).

| **Confounder** | **n** | **Univariate** | | | **Multivariate** | | |
| --- | --- | --- | --- | --- | --- | --- | --- |
|  |  | **OR** | **p** | **95% CI** | **OR** | **p** | **95% CI** |
| Age per year | 299 | 1.012 | 0.330 | 0.988-1.036 | - | - | - |
| Hypertonia yes vs. no | 102 | 1.284 | 0.356 | 0.765-2.181 | - | - | - |
| Diabetes yes vs. no | 27 | 1.984 | 0.099 | 0.879-4.794 | - | - | - |
| Heart disease  yes vs. no | 37 | 1.160 | 0.700 | 0.545-2.472 | - | - | - |
| ASA III/IV vs. I/II | 158 | 1.552 | 0.100 | 0.920-2.618 | - | - | - |
| Neoadjuvant chemotherapy  yes vs. no | 42 | 0.495 | 0.107 | 0.210-1.163 | - | **-** | - |
| SCS groups  high vs. intermediate | 220 | 2.033 | **0.031** | **1.067-3.875** | - | - | - |
| Small bowel resection | 77 | 1.822 | **0.035** | **1.043-3.182** | 0.954 | 0.888 | 0.493-1.834 |
| Large bowel resection | 120 | 3.170 | **< 0.001** | **1.870-5.375** | 2.708 | **0.002** | **1.449-5.061** |
| Rectosigmoidectomy | 174 | 1.411 | 0.200 | 0.833-2.390 | - | - | - |
| LNE pelvic | 244 | 0.885 | 0.712 | 0.463-1.691 | - | - | - |
| LNE paraaortal | 244 | 0.795 | 0.481 | 0.419-1.506 | - | - | - |
| Abdominal peritoneum stripping | 192 | 2.209 | **0.007** | **1.236-3.948** | 1.627 | 0.120 | 0.881-3.004 |
| Diaphragm stripping | 221 | 1.012 | 0.969 | 0.565-1.809 | - | - | - |
| Splenectomy | 20 | 2.930 | **0.022** | **1.171-7.329** | 2.127 | 0.122 | 0.818-5.533 |
| Liver resection | 25 | 1.578 | 0.298 | 0.668-3.729 | - | - | - |
| Ileostoma vs. no stoma | 16 | 1.029 | 0.962 | 0.319-3.317 | - | - | - |
| Kolostoma vs. no stoma | 50 | 2.058 | **0.027** | **1.086-3.903** | - | **-** | - |
| Only small bowel | 4 | 0 | 0.999 | - | - | - | - |
| Only large bowel | 17 | 3.823 | **0.020** | **1.232-11.864** | - | - | - |
| Only rectosigmoid | 82 | 1.344 | 0.471 | 0.601-3.008 | - | - | - |
| Small+Large bowel | 20 | 4.469 | **0.006** | **1.547-12.910** | - | -- | - |
| Recto+Large bowel | 40 | 4.037 | **0.002** | **1.705-9.556** | - | - | - |
| Recto+Small bowel | 10 | 2.341 | 0.259 | 0.535-10.242 | - | - | - |
| All three bowel res. | 43 | 3.236 | **< 0.001** | **1.376-7.614** | - | - | - |

**Supplementary Table S4:** Univariate Cox regression analyses of factors impacting on long term survival in ovarian cancer patients with FIGO IIIC or IVA/B ovarian cancer after maximum effort debulking surgery (n=263). Patients were included if follow up beyond three months was available. HR, hazard ratio; CI, confidence interval. *Data on adjuvant chemotherapy were only available for 152 patients and cases with NACT were excluded, resulting in 135 cases included in the analysis of the impact of time to adjuvant chemotherapy on long term outcome.

|  | **Progression free survival** | | | **Overall survival** | | |
| --- | --- | --- | --- | --- | --- | --- |
|  | **HR** | **p-value** | **95% CI** | **HR** | **p-value** | **95% CI** |
| **Age per year** | 1.018 | **0.005** | **1.006-1.031** | 1.024 | **0.001** | **1.010-1.038** |
| **Cytoreductive surgery complete vs. incomplete** | 0.583 | **0.005** | **0.401-0.849** | 0.639 | **0.030** | **0.426-0.958** |
| **Neoadjuvant chemotherapy** | 0.905 | 0.610 | 0.617-1.327 | 0.957 | 0.832 | 0.639-1.433 |
| **Time to adjuvant chemotherapy > 6 weeks vs. < 6 weeks*** | 0.844 | 0.382 | 0.577-1.235 | 0.851 | 0.445 | 0.563-1.287 |
| **Surgical complexity score high (8-16) vs. Intermediate (4-7)** | 1.369 | 0.053 | 0.996-1.883 | 1.228 | 0.232 | 0.877-1.718 |
| **Pelvic lymphadenectomy** | 0.845 | 0.371 | 0.585-1.222 | 0.915 | 0.668 | 0.611-1.371 |
| **Para-aortic**  **lymphadenectomy** | 0.735 | 0.091 | 0.514-1.050 | 0.783 | 0.216 | 0.531-1.154 |
| **Diaphragm**  **stripping/resection** | 1.379 | 0.061 | 0.986-1.928 | 1.246 | 0.218 | 0.878-1.770 |
| **Abdominal peritoneum**  **stripping** | 1.521 | **0.005** | **1.133-2.041** | 1.486 | **0.013** | **1.088-2.029** |
| **Recto-sigmoidectomy/**  **anastomosis** | 0.941 | 0.664 | 0.713-1.240 | 0.899 | 0.478 | 0.670-1.206 |
| **Large bowel resection/s** | 1.271 | 0.098 | 0.956-1.690 | 1.394 | **0.032** | **1.029-1.887** |
| **Small-bowel resection/s** | 1.733 | **<0.001** | **1.272-2.362** | 2.029 | **< 0.001** | **1.465-2.811** |
| **Liver resection/s** | 1.335 | 0.245 | 0.820-2.172 | 1.681 | 0.050 | 1.001-2.824 |
| **Splenectomy** | 1.466 | 0.169 | 0.850-2.528 | 1.137 | 0.667 | 0.633-2.044 |
| **Number of removed LN in patients with pelvic AND/OR paraaortic lymphadenectomy** | 0.996 | 0.120 | 0.991-1.001 | 0.997 | 0.209 | 0.991-1.002 |
| **Pathologically confirmed N1 after pelvic AND/OR paraaortic LNE (131/176)** | 1.458 | 0.066 | 0.976-2.256 | 2.178 | 0.317 | 0.812-1.900 |

**Supplementary Table S5:** Progression free survival and overall survival depending on necessity for small-bowel resection during maximum effort debulking surgery. Patients were included (n=225) if they had FIGO IIIC or IVA/B ovarian cancer, complete tumour resection and follow up beyond three months.

|  | | **Small-bowel resection** | | **p-value**  (Log Rank) |
| --- | --- | --- | --- | --- |
|  |  | **No (n=172)** | **Yes (n=53)** |  |
| **Progression free survival** | 3-year rate (%) | 37.0 | 22.7 | **< 0.001** |
|  | 5-year rate (%) | 26.7 | 11.5 |  |
|  | Median (months [95%CI]) | 26.9 [22.1-31.6] | 16.8 [14.1-19.5] |  |
| **Overall survival** | 3-year rate (%) | 57.0 | 31.8 | **< 0.001** |
|  | 5-year rate (%) | 34.7 | 16.3 |  |
|  | Median (months [95%CI]) | 42.1 [35.3-48.9] | 19.3 [11.8-26.8] |  |

**Supplementary Table S6:** Median progression free survival (PFS) and overall (OS) depending on number and region of bowel resection/s during primary maximum effort debulking surgery, calculated with the Kaplan-Meier method. Patients were included (n=225) if they had FIGO IIIC or IVA/B ovarian cancer, complete tumor resection and follow up beyond three months. P-values according to Log Rank test.

| **Region/s with bowel resection/s** | **N** | **Progression free survival** | | | | **Overall survival** | | | |
| --- | --- | --- | --- | --- | --- | --- | --- | --- | --- |
|  |  | **3-year rate [%]** | **Median [Months]** | **SE** | **p-value** | **3-year rate [%]** | **Median [Months]** | **SE** | **p-value** |
| **No bowel resections** | **70** | 38.3 | 26.2 | 4.9 | ref. | 59.4 | 46.0 | 5.7 | ref. |
| **Rectosigmoid AND Colon** | **30** | 40.8 | 29.4 | 4.4 | 0.515 | 55.4 | 49.3 | 10.7 | 0.575 |
| **Small Bowel OR Rectosigmoid OR Colon** | **75** | 35.6 | 25.5 | 3.4 | 0.448 | 55.8 | 38.7 | 5.0 | 0.815 |
| **Small bowel AND (Rectosigmoid AND/OR Colon)** | **17** | 32.7 | 17.2 | 3.2 | **0.028** | 44.0 | 24.6 | 5.7 | 0.062 |
| **Ileocoecal**  **(72.7% in comb. with rectosigmoid)** | **33** | 15.2 | 14.5 | 2.2 | **0.001** | 24.2 | 19.2 | 3.6 | **<0.001** |


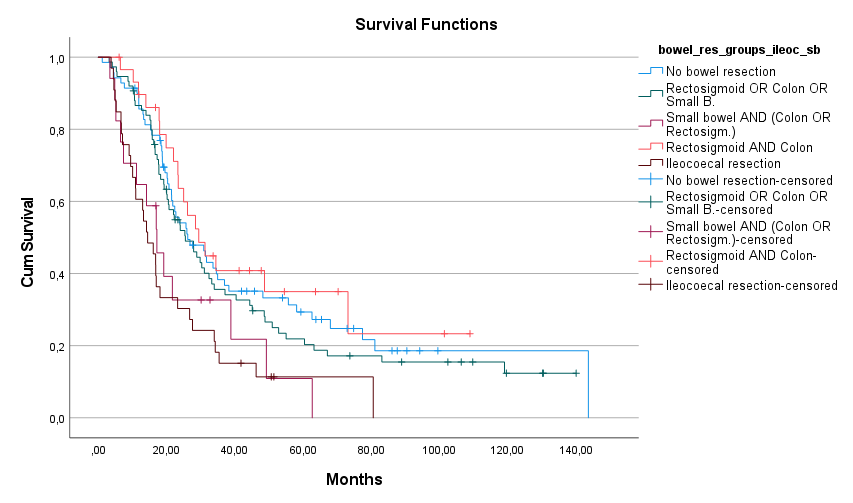
**Supplementary Figure S7:** Kaplan-Meier curve of progression free survival (PFS) depending on number and region of bowel resection during primary maximum effort debulking surgery. Patients were included (n=225) if they had FIGO IIIC or IVA/B ovarian cancer, complete tumor resection and follow up beyond three months.


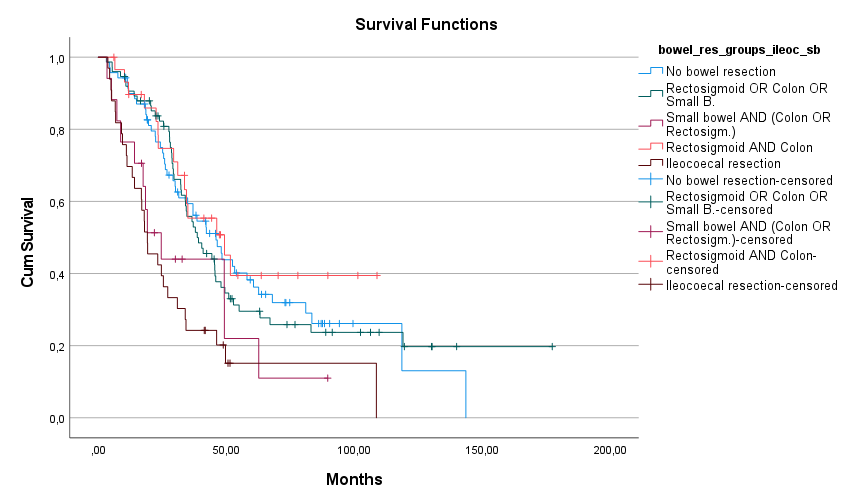
**Supplementary Figure S8:** Kaplan-Meier curve of overall survival (OS) depending on number and region of bowel resection during primary maximum effort debulking surgery. Patients were included (n=225) if they had FIGO IIIC or IVA/B ovarian cancer, complete tumor resection and follow up beyond three months.

**Supplementary Table S9:** Peritoneal Carcinoma Index (PCI) in patients with different number and regions of bowel resection/s during primary maximum effort debulking surgery. PCI was available only for a proportion of patients analysed for long-term outcome (Supplementary Table S3). Patients were included if they had FIGO IIIC or IVA/B ovarian cancer, complete tumor resection and follow up beyond three months. Because of non-parametric distribution of data, central trends were analysed with Kruskal-Wallis test, yielding a p-value of < 0.001. P-values of post-hoc pairwise testing are shown in the table.

| **Region/s with bowel resection/s** | **N**  (missing) | **PCI,**  **Mean [SD]** | **p-value** | | | | |
| --- | --- | --- | --- | --- | --- | --- | --- |
| No bowel resections | **42** (28) | 11.7 [6.4] | Ref. |  |  |  |  |
| Rectosigmoid AND Colon | **28** (2) | 18.8 [4.9] | **< 0.001** | Ref. |  |  |  |
| Small Bowel  OR Rectosigmoid  OR Colon | **48** (27) | 21.7 [7.5] | **< 0.001** | 0.134 | Ref. |  |  |
| Small bowel AND  (Rectosigmoid AND/OR Colon) | **12** (5) | 20.3 [7.6] | **< 0.001** | 0.529 | 0.666 | Ref. |  |
| Ileocoecal  (72.7% in comb. with rectosigmoid) | **22** (11) | 20.4 [6.2] | **< 0.001** | 0.533 | 0.487 | 0.912 | Ref. |

**Supplementary Table S10:** Peritoneal Carcinoma Index (PCI) of intestinal Regions (9-12) in patients with different number and regions of bowel resection/s during primary maximum effort debulking surgery. PCI was available only for a proportion of patients analysed for long-term outcome (Supplementary Table S3). Patients were included if they had FIGO IIIC or IVA/B ovarian cancer, complete tumor resection and follow up beyond three months. Because of non-parametric distribution of data, central trends were analysed with Kruskal-Wallis test, yielding a p-value of < 0.001. P-values of post-hoc pairwise testing are shown in the table.

| **Region/s with bowel resection/s** | **N**  (missing) | **PCI 9-12,**  **Mean [SD]** | **p-value** | | | | |
| --- | --- | --- | --- | --- | --- | --- | --- |
| No bowel resections | **42** (28) | 1.5 [2.4] | Ref. |  |  |  |  |
| Rectosigmoid AND Colon | **28** (2) | 4.5 [3.1] | **< 0.001** | Ref. |  |  |  |
| Small Bowel  OR Rectosigmoid  OR Colon | **48** (27) | 5.3 [3.6] | **< 0.001** | 0.431 | Ref. |  |  |
| Small bowel AND  (Rectosigmoid AND/OR Colon) | **12** (5) | 6.8 [2.7] | **< 0.001** | 0.051 | 0.132 | Ref. |  |
| Ileocoecal  (72.7% in comb. with rectosigmoid) | **22** (11) | 6.3 [3.3] | **< 0.001** | 0.076 | 0.218 | 0.637 | Ref. |

**Supplementary Table S11:** Surgical Complexity Score (SCS) in patients with different number and regions of bowel resection/s during primary maximum effort debulking surgery. Patients were included (n=225) if they had FIGO IIIC or IVA/B ovarian cancer, complete tumor resection and follow up beyond three months. Because of non-parametric distribution of data, central trends were analysed with Kruskal-Wallis test, yielding a p-value of < 0.001. P-values of post-hoc pairwise testing are shown in the table.

| **Region/s with bowel resection/s** | **N** | **SCS,**  **Mean [SD]** | **p-value** | | | | |
| --- | --- | --- | --- | --- | --- | --- | --- |
| No bowel resections | **70** | 6.6 [1.5] | Ref. |  |  |  |  |
| Rectosigmoid AND Colon | **30** | 12.7 [1.5] | **< 0.001** | Ref. |  |  |  |
| Small Bowel  OR Rectosigmoid  OR Colon | **75** | 10.3 [1.6] | **< 0.001** | **< 0.001** | Ref. |  |  |
| Small bowel AND  (Rectosigmoid AND/OR Colon) | **17** | 11.7 [2.1] | **< 0.001** | 0.239 | **0.044** | Ref. |  |
| Ileocoecal  (72.7% in comb. with rectosigmoid) | **33** | 12.6 [2.1] | **< 0.001** | 0.922 | **< 0.001** | 0.265 | Ref. |
